# Active Chitosan Films Enriched with Yerba Mate Kombucha Infusion: Formulation and Characterization

**DOI:** 10.3390/ijms27125346

**Published:** 2026-06-13

**Authors:** Celeste Cottet, Pamela A. Kikot, Matías L. Nobile, Marcela F. Almassio, Andrés G. Salvay, Mercedes A. Peltzer

**Affiliations:** 1 Laboratory of Obtention, Modification, Characterization and Evaluation of Materials (LOMCEM), Department of Science and Technology, National University of Quilmes, Bernal B1876BXD, Argentina; asalvay@unq.edu.ar; 2 National Scientific and Technical Research Council (CONICET), Buenos Aires C1033AAG, Argentina; pamela.kikot@unq.edu.ar (P.A.K.); mnobile@unq.edu.ar (M.L.N.); 3 Biotechnological Materials Laboratory (LaMaBio), Department of Science and Technology, National University of Quilmes, Bernal B1876BXD, Argentina; 4 Biotransformation and Nucleic Acids Laboratory (LaByQAN), Department of Science and Technology, National University of Quilmes, Bernal B1876BXD, Argentina; 5 INQUISUR, Department of Chemistry, National University of the South (UNS)-CONICET, Bahía Blanca B8000FTN, Argentina; almassio@criba.edu.ar; 6 Commission for Scientific Research (CIC-PBA), Buenos Aires B1900TOL, Argentina

**Keywords:** active packaging, food contact materials, antioxidant materials, bio-based materials, natural plasticization

## Abstract

The development of bio-based active packaging materials has gained increasing attention as a sustainable alternative to synthetic plastics. In this study, chitosan-based films incorporating yerba mate kombucha infusion (YMK-I) were developed and fully characterized. Films were prepared using different YMK-I concentrations (25–100% *v*/*v*) as solvent, with acetic acid-based chitosan films as controls. The infusion showed pH 2.5, titratable acidity of 3.5%, total solids of 6%, high phenolic content (1085 mg GAE/L), and reducing sugars (18.3 g/L). Acetic and lactic acids were identified by high-performance liquid chromatography (HPLC). Minimum Inhibitory Concentration (MIC) values ranged from 0.03 µg/mL for *Staphylococcus aureus* to 0.3 µg/mL for *Escherichia coli* and *Pseudomonas aeruginosa*. Rheological results indicated that YMK-I performed similarly to acetic acid as a solvent. Fourier Transformed Infrared with Attenuated Total Reflectance (FTIR-ATR) suggested interactions between chitosan and bioactive compounds. Thermal analyses showed that YMK-I acted as a plasticizer and introduced thermolabile components, altering glass transition and degradation behavior. Increasing YMK-I content reduced tensile strength and increased elongation, indicating greater flexibility, while water vapor permeability increased due to hydrophilic compounds. Films enriched with YMK-I exhibited high antioxidant activity (Radical Scavenging Activity > 85%) and strong antimicrobial effects (>98% inhibition) against *E. coli* and *S. aureus*. These results highlight the potential of chitosan–kombucha films as multifunctional materials for specialized applications.

## 1. Introduction

In recent years, the development of bio-based active packaging materials has gained increasing attention as a sustainable alternative to synthetic plastics, particularly in food preservation systems. Among the most widely studied biopolymers, chitosan, a natural polysaccharide derived from the deacetylation of chitin, exhibits remarkable properties such as biodegradability, film-forming ability, and inherent antimicrobial activity, making it a suitable candidate for functional packaging applications [1,2,3]. In addition to food packaging systems, polymeric films based on natural polymers have attracted interest for a broad range of applications in biomedical, pharmaceutical, and cosmetic fields due to their biocompatibility and tunable functional properties. In food applications, active films can improve product preservation through controlled release of bioactive compounds and inhibition of microbial growth, while in biomedical systems they have been proposed as wound dressings, drug delivery platforms, and tissue engineering materials because of their ability to provide a favorable microenvironment and incorporate bioactive agents [4,5,6,7].

Furthermore, the increasing environmental concerns associated with the accumulation of non-degradable petroleum-based plastics have intensified the search for biodegradable alternatives. Unlike conventional synthetic materials, biopolymeric films can undergo degradation through hydrolytic, enzymatic, and microbial processes, leading to the formation of environmentally compatible compounds that can be naturally reintegrated into ecological cycles [8,9]. The biodegradation behavior of these materials depends on several factors, including polymer composition, molecular structure, crystallinity, and environmental conditions [10]. Consequently, the use of biodegradable films not only contributes to reducing environmental impact but also aligns with current strategies promoting sustainable material development and circular economy principles.

Chitosan is a weak base and does not dissolve in water or organic solvents; however, it becomes soluble in diluted acidic aqueous solutions, below pH 6. This solubility is due to the amine groups in its molecular structure, which become protonated in acidic environments, generating positive charges along the polymer chains (-NH3^+^) [11,12]. Chitosan can be effectively dissolved using a range of organic and inorganic acids, such as acetic, formic, L-glutamic, lactic, hydrochloric, and malic acids. Many organic acids, which are naturally present in fruits and fermented foods, not only facilitate the dissolution of chitosan but also possess antimicrobial properties against foodborne pathogens [13,14]. In this context, kombucha, an acidic beverage produced by fermenting a sugared infusion with a symbiotic culture of bacteria and yeasts (SCOBY), has emerged as a novel compound of interest. The SCOBY is a microbial consortium is a structured community of multiple species of microorganisms that interact symbiotically [15,16], establishing interdependent metabolic relationships that enable the survival and functionality of the complete system [17,18]. In these consortia, microorganisms do not operate in isolation but rather depend on syntrophic interactions where the metabolic products of some species constitute essential substrates for others [16,18,19]. In particular, the kombucha SCOBY constitutes a microbial consortium of low taxonomic diversity but high functional complexity. Were dominant bacteria are those from genus Komagataeibacter (including species such as *K. rhaeticus*, *K. xylinus*, and *K. intermedius*). Additionally, other genera of acetic acid bacteria from the family Acetobacter might be detected (Gluconobacter or Acetobacter). Then, is possible to find dominant yeast such as Brettanomyces (Dekkera) bruxellensis and species of the genus Zygosaccharomyces (including *Z. bailii* and *Z. parabailii*) as the most abundant fermentative fungi in the consortium. Finally deoending on the geographic origin of kombucha, SCOBY type and the sampled fraction studies (pellicle versus liquid), lactic acid bacteria and additional yeast could be found [20,21,22].

Considering its acidity of kombucha beverage, it could serve as an alternative solvent for chitosan, offering a natural substitute for conventional acids [23]. Additionally, kombucha provides bioactive compounds that may enhance the functional properties of the chitosan matrix.

Kombucha infusion contains organic acids, minerals, and vitamins primarily derived from yerba mate, as well as amino acids and biologically active compounds, particularly polyphenols [24]. The bioactive potential of the fermented infusion is largely attributed to these compounds, which have been widely reported to exhibit strong antioxidant and antimicrobial properties [25,26].

Yerba mate is a South American plant traditionally consumed as an infusion. It is rich in phenolic compounds, such as chlorogenic acids, flavonoids (quercetin, kaempferol), and xanthines (caffeine, theobromine), which contribute to its potent bioactivity [24,27]. When fermented into kombucha, yerba mate undergoes biochemical transformations that may enhance the extractability and bioavailability of its antioxidant constituents [28]. The incorporation of yerba mate kombucha infusion into chitosan matrices is therefore a promising strategy to develop multifunctional films with improved physicochemical and biological properties.

Previous studies have explored the incorporation of natural extracts into biopolymeric matrices, reporting improvements in mechanical flexibility, oxidative stability, and microbial resistance of the resulting films [29,30]. Nevertheless, the use of kombucha, particularly derived from yerba mate, as both a solvent and bioactive additive for chitosan film formation remains largely unexplored.

This work aims to develop and characterize chitosan-based films formulated with different concentrations of Yerba mate kombucha infusion (YMK-I), focusing on their structural, thermal, mechanical, barrier, optical, antioxidant, and antimicrobial properties. The incorporation of YMK-I is expected not only to enhance the functionality of the films but also to contribute to the valorization of traditional plant-based infusions in sustainable packaging technologies.

## 2. Results and Discussion

### 2.1. Characterization of YMK-I

The yerba mate kombucha infusion (YMK-I) showed a total solids content of 6% (g solids/100 g of infusion), indicating the presence of dissolved and suspended compounds generated during fermentation. The pH was 2.5, reflecting a markedly high acidity level. This pronounced acidity is likely due to the extended fermentation period of 21 days, longer than the typical 10-day fermentation applied to kombucha intended for direct consumption [31,32]. Furthermore, the titratable acidity reached 3.5% (g acetic acid/100 mL sample), confirming the strong acidic profile of the infusion and its potential relevance for preservation and antimicrobial activity [33]. After fermentation, the kombucha infusion presented a total phenolic content of 1085 ± 4 mg GAE (gallic acid equivalents)/L, mainly derived from the yerba mate used for its preparation. This value is consistent with those reported in previous studies on yerba mate infusions under comparable conditions [34]. In addition, similar values of phenolic compounds were reported for blank tea kombucha describing values of 1.09 mg GAE/mL and lower values for green tea kombucha (0.7 mg GAE/mL) [35]. In parallel, the reducing sugar content was 18.3 ± 3.6 g glucose/L, which was within the expected value due to microbial consumption, confirming the metabolic activity of the symbiotic culture throughout the fermentation period. These results demonstrate the biochemical modifications occurring during the fermentation [36]. Kombucha contains a wide range of organic acids and its composition can vary depending on factors such as the starter culture, sugar and tea concentrations, fermentation time, and temperature. Commonly reported acids include acetic, gluconic, glucuronic, citric, L-lactic, malic, tartaric, malonic, oxalic, succinic, and pyruvic acids [24,35,37]. Acetic and lactic acids were detected and quantified in the YMK-I under the experimental conditions employed, as shown in Table 1. The separation enabled the identification of both acids, which were resolved according to their characteristic retention times. The obtained profile reflects the contribution of fermentation-derived organic acids to the acidic composition of the system, in agreement with the expected chemical characteristics of kombucha-based infusions.

The minimum inhibitory concentration (MIC) values for YMK-I ranged from 0.03 µg/mL for *S. aureus* to 0.3 µg/mL for *E. coli* and *P. aeruginosa*. This difference is largely driven by structural variations in their cell walls and membranes. In this context, Gram-negative bacteria exhibited higher resistance than Gram-positive bacteria, primarily due to their more complex cell envelope. The outer membrane of Gram-negative bacteria rich in lipopolysaccharides, absent in Gram-positive species, serves as an effective permeability barrier, limiting the entry of many antimicrobial agents and contributing to their increased resistance [38,39]. In addition to cell wall structural differences, the antimicrobial activity of YMK-I may also be associated with the combined action of bioactive compounds naturally present in yerba mate and metabolites generated during kombucha fermentation. Yerba mate contains several compounds with reported antimicrobial activity, including polyphenols, flavonoids, and xanthines, while the fermentation process additionally produces bioactive metabolites such as organic acids and other secondary compounds that may contribute synergistically to microbial growth inhibition [25,40,41,42]. Organic acids, particularly acetic acid, may disrupt membrane integrity and intracellular pH homeostasis, while phenolic compounds naturally present in yerba mate can alter membrane permeability and interfere with microbial metabolic pathways [43,44]. The synergistic interaction among these compounds likely contributes to the low MIC values observed in the present study.

### 2.2. Characterization of Film Forming Dispersions and Active Films

#### 2.2.1. Rheological Behavior of Film Forming Dispersions

**Figure 1:**
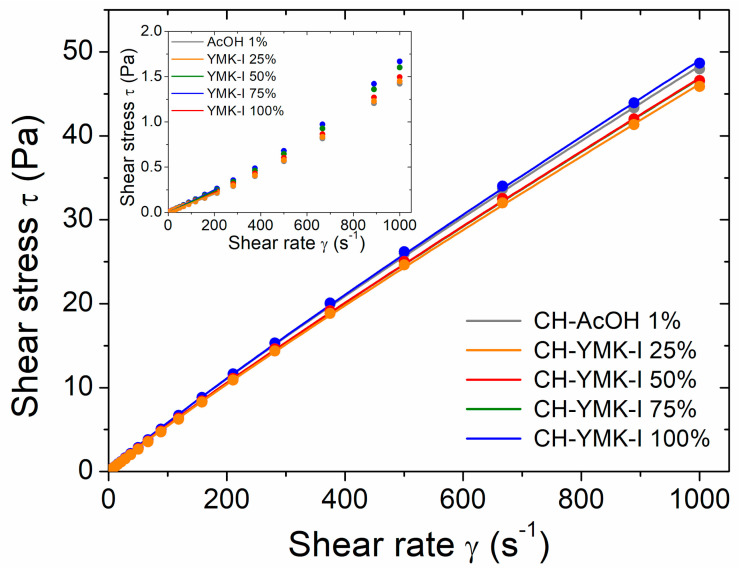
Rotational rheology of dissolutions of chitosan in acetic acid dilutions and in various dilutions of yerba mate kombucha tea. The acetic acid and yerba mate kombucha tea dilutions used to dissolve chitosan are shown in the insert of the figure. Experimental data were fitted with the Herschel–Bulkley model (Equation (1)). Fitted parameters are shown in Table 3.

The shear stress τ as a function of the shear rate γ for chitosan dissolved in acetic acid dilutions and in various dilutions of YMK-I is shown in Figure 1. Experimental data were fitted to the Herschel–Bulkley model (Equation (2)), and the parameters obtained from the fitting are presented in Table 2. The reported R^2^ values, being close to 1, indicate a good fit of the model across the entire range of shear rates studied.

All chitosan dissolutions exhibited pseudoplastic behaviour (n < 1). As shown in Table 2, this shear-thinning behaviour was practically identical for all these samples. Similar pseudoplastic performance has also been reported for chitosan dispersions prepared in 1% (*v*/*v*) acetic acid and mechanically stirred at 500 rpm for 2 h [45]. As observed in Figure 1, the shear stress vs. shear rate curves were very similar among all samples, reaching maximum shear stress values at the end of the test (γ = 1000 s^−1^), ranging from lowest to highest as follows: 45.9 Pa (CH-YMK-I 25%), 46.6 (CH-YMK-I 50% and CH-YMK-I 55%), 48.0 (CH-AcOH 1%) and 48.7 Pa (CH-YMK-I 100%). Moreover, the consistency index K, directly related to the apparent viscosity, was nearly identical for the CH-AcOH 1% and CH-YMK-I 100% samples, and slightly lower for the remaining chitosan dissolutions (Table 2).

**Figure 2:**
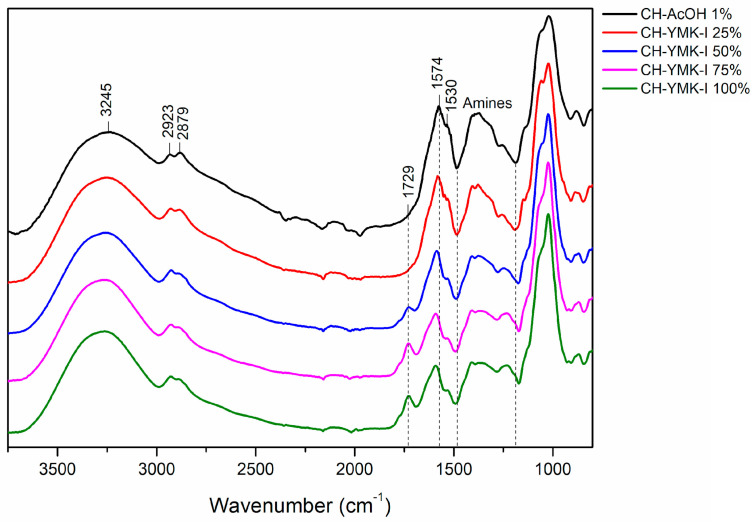
Fourier Transform Infrared Spectrum with Attenuated Total Reflectance (FTIR-ATR) of chitosan and kombucha-based films.

In addition, the dilutions used to dissolve the chitosan were also analysed (see insert of Figure 2). These dilutions exhibited considerably lower shear stress values than the chitosan dissolutions and showed deviations from linearity at shear rates below 5 s^−1^ and above 200 s^−1^, mimicking a false dilating behaviour. These deviations were likely caused by torque noise at low speeds and by turbulence or fluid inertia effects at high speeds, typical of low viscosity fluids. Therefore, analysis were performed within the shear rate range of 5 s^−1^ to 200 s^−1^. As shown in Table 2, all dilutions exhibited matching newtonian behaviour (n ≈ 1), with consistency index K values ranging from 9.0 ± 0.6 × 10^−4^ Pa.s (AcOH 1%) to 11.8 ± 0.8 × 10^−4^ Pa.s (YMK-I 100%). As can be seen in Figure 2, the final shear stress values reached by the chitosan dissolutions were approximately 1.3 orders of magnitude higher than those of the acetic acid and yerba mate kombucha infusion dilutions. Furthermore, K values obtained for chitosan dissolutions were approximately 2.4 orders of magnitude higher than those of the corresponding solvent dilutions.

#### 2.2.2. Fourier Transform Infrared Spectroscopy with Attenuated Total Reflectance (FTIR-ATR)

The infrared spectra of chitosan and kombucha-based films allowed for the analysis of their composition by associating absorption bands with specific functional groups. Figure 2 shows the FTIR results for chitosan and different YMK-I concentration films. As expected, the control film (CH-AcOH 1%) spectrum was very similar to previous studies [23]. For all films, a broad band at 3245 cm^−1^ corresponds to stretching vibrations of N-H and O-H bonds, indicating the presence of hydroxyl and amine groups in the film structure. The C–H bond of the –CH_2_ and –CH_3_ were observed at 2932 and 2879 cm^−1^, respectively [46].

Additionally, a band at 1729 cm^−1^ became progressively more intense with increasing YMK-I concentration. This absorption band can be assigned to C=O stretching vibrations of carboxylic acid groups [47]. Since YMK-I contains organic acids generated during fermentation, including acetic and other fermentation-derived acids, the increase in intensity may be associated with their higher incorporation into the polymeric matrix. This was the first evident difference between the control film and YMK-I-containing films.

The bands observed at 1574 and 1530 cm^−1^ were attributed mainly to amide II vibrations and N–H bending associated with residual acetylated groups and amino functionalities of chitosan [48]. Contributions from aromatic ring vibrations of phenolic compounds naturally present in yerba mate may also overlap in this region. A slight shift in these bands toward higher wavenumbers in YMK-I-containing films compared to pure chitosan suggests the formation of intermolecular interactions, likely hydrogen bonding between chitosan functional groups and bioactive compounds present in YMK-I.

The C-N bond of primary, secondary and tertiary amines were detected between 1200 and 1400 cm^−1^. Also the C-O-C bond of saccharide ring was observed at 1022 cm^−1^ [49].

#### 2.2.3. Scanning Electron Microscopy (SEM)

**Figure 3:**
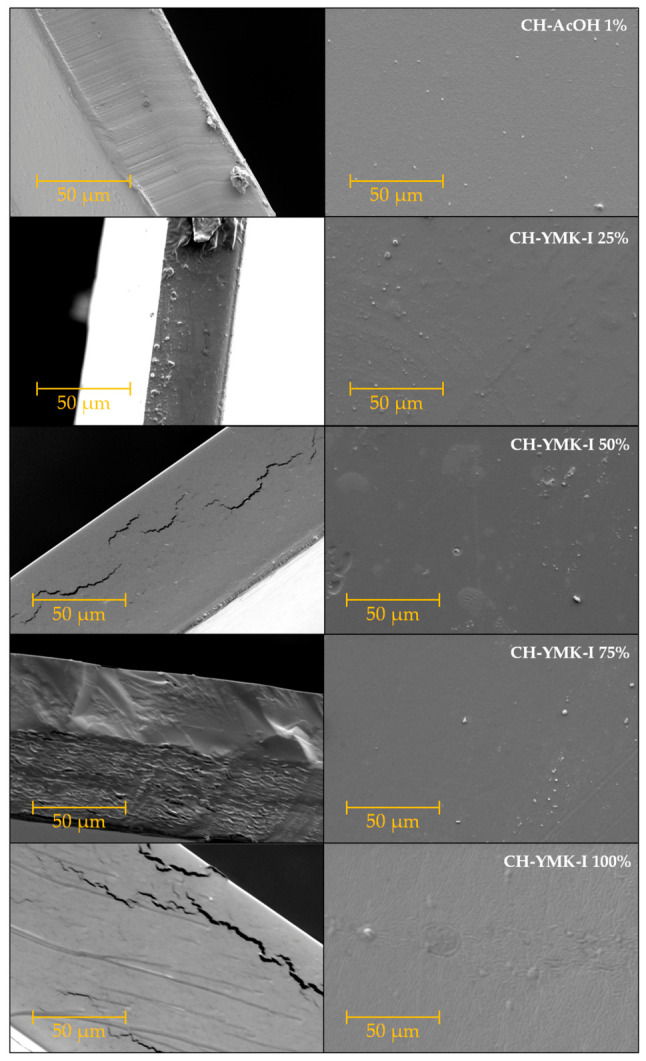
SEM images of the surfaces and cross sections of chitosan and kombucha-based films (magnification 1500×). The images on the left correspond to the thickness measurements, while the images on the right show the surface morphology of the films.

Chitosan films were uniform, flexible, smooth, and no cracks or pores were observed visually. Figure 3 shows SEM images of the prepared films. Scanning electron microscopy was used to examine the morphology of the chitosan and kombucha-based films.

All samples exhibited a non-porous structure, and the incorporation of the kombucha infusion did not compromise the material’s homogeneity, indicating good compatibility and integrity among the film components. However, increasing the concentration of yerba mate kombucha infusion in the formulation led to a slight roughness on the surface, which reduced the smoothness and uniformity of the final films. Nevertheless, this roughness did not affect the quality of the films.

#### 2.2.4. Differential Scanning Calorimetry (DSC)

**Figure 4:**
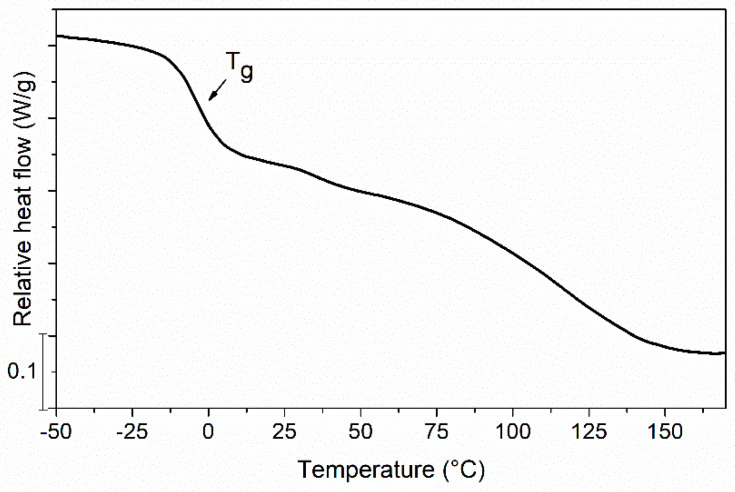
DSC Thermogram of YMK-I.

DSC analysis was conducted to characterize the thermal transitions, both for yerba mate kombucha infusion and chitosan films. The DSC curves for the dried kombucha solids exhibited a single glass transition (Tg) at −5.37 °C as it is showed in Figure 4. There are very few studies reporting the thermal transitions of kombucha by DSC; however, the Tg obtained in this work is close to a value previously reported in the literature, supporting the reliability of the present findings [50].

**Figure 5:**
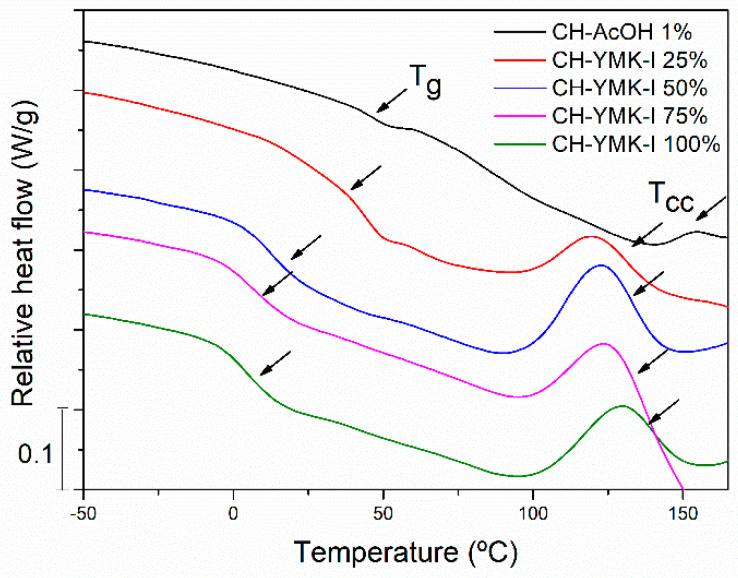
DSC Thermograms of chitosan and kombucha-based films.

The glass transition temperature (Tg) plays a critical role in defining the thermal behavior of biopolymer-based materials. In this work, the effect of the solvent used during film preparation on the Tg of chitosan films was examined. Thermograms of chitosan and kombucha based films are presented in Figure 5 and the corresponding transition temperatures are summarized in Table 3. The DSC curves of all films exhibited a single Tg, with values ranging from 7 to 53 °C depending on the kombucha infusion concentration. This parameter is commonly used to assess the miscibility between film components. In a fully miscible system, a single Tg is typically observed in the DSC thermograms, indicating homogeneous phase behavior. The highest Tg was observed in the control films, which is consistent with values reported in previous studies for similar materials [51,52]. A clear decrease in Tg was observed with increasing concentrations of YMK-I, likely due to the plasticizing effect of the infusion. The compounds present in the infusion may disrupt polymer–polymer interactions, enhancing molecular mobility. Consequently, the reduction in intermolecular forces between polymer chains increases the free volume and overall system flexibility, shifting the Tg to lower temperatures [53].

**Table 3:** Transition thermal temperatures of chitosan and kombucha-based films. Temperature error: ±1 °C.

Sample	Tg (°C)	Tcc (°C)	ΔHcc (J/g)
CH-AcOH 1%	53	154	1.97
CH-YMK-I 25%	47	120	12
CH-YMK-I 50%	19	120	23
CH-YMK-I 75%	12	125	32
CH-YMK-I 100%	7.8	127	24

An exothermic event was observed across all formulations, is most likely associated with a cold crystallization process, wherein previously amorphous polymer chains gain sufficient mobility upon heating to reorganize into more ordered crystalline structures [54]. Moreover, the cold crystallization exothermic peak (Tcc) shifted to lower temperatures with increasing amounts of YMK-I in the formulation, suggesting a faster crystallization rate. Additionally, the increase in the enthalpy of cold crystallization (∆Hcc) indicates a higher degree of crystallinity in the chitosan matrix. This behavior may also be attributed to the presence of low-molecular-weight compounds in the kombucha, which could act as natural plasticizers, enhancing polymer chain mobility and promoting more efficient crystalline organization during heating [55].

#### 2.2.5. Thermogravimetric Analysis (TGA)

**Figure 6:**
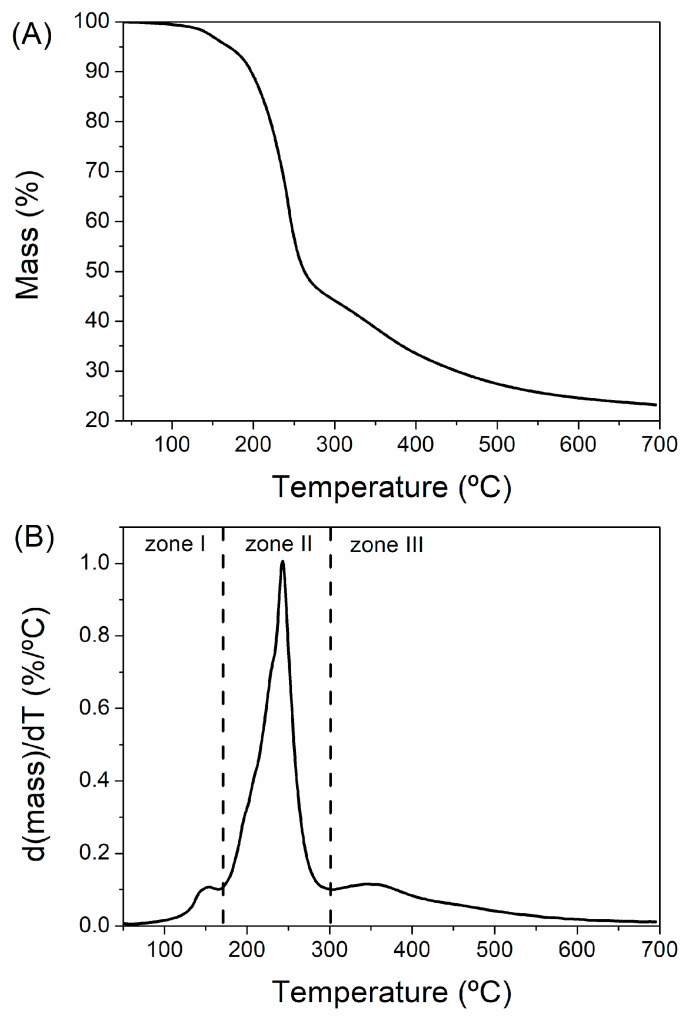
Thermogravimetric Curves of yerba mate kombucha infusion (YMK-I): (**A**) mass loss as a function of temperature and (**B**) Derivative of the percentage mass as a function of temperature.

Figure 6 shows the degradation profile of kombucha infusion solids. It is possible to see that kombucha degrades in a multistep profile, which the DTG curve describes clearly. The initial degradation temperature after water loss (T_i_) was determined as the temperature at which 10% of mass loss occurs, while the temperatures of thermal degradation (T_max_) for each stage were determined from the peaks of the derivative curves (DTG).

DTG curves revealed three distinct degradation zones: the first zone (T_max1_ = 150 °C), observed between 40 and 175 °C, accounted for approximately 5% mass loss and was primarily associated with moisture evaporation. The second zone (T_max2_ = 243 °C), spanning from 175 to 300 °C and representing nearly 50% mass loss, corresponded to the thermal decomposition of yerba mate infusion constituents, including xanthines (e.g., caffeine and theobromine), reducing sugars, and organic acids. The third zone (T_max3_ = 350 °C), occurring above 300 °C and responsible for approximately 21% mass loss, was attributed to higher-molecular-weight and more thermally stable compounds. At 700 °C, the sample the yielded a small amount of residual ash (approximately 24%). To the best of our knowledge, no studies have previously evaluated the thermal stability of yerba mate kombucha infusion by TGA.

**Figure 7:**
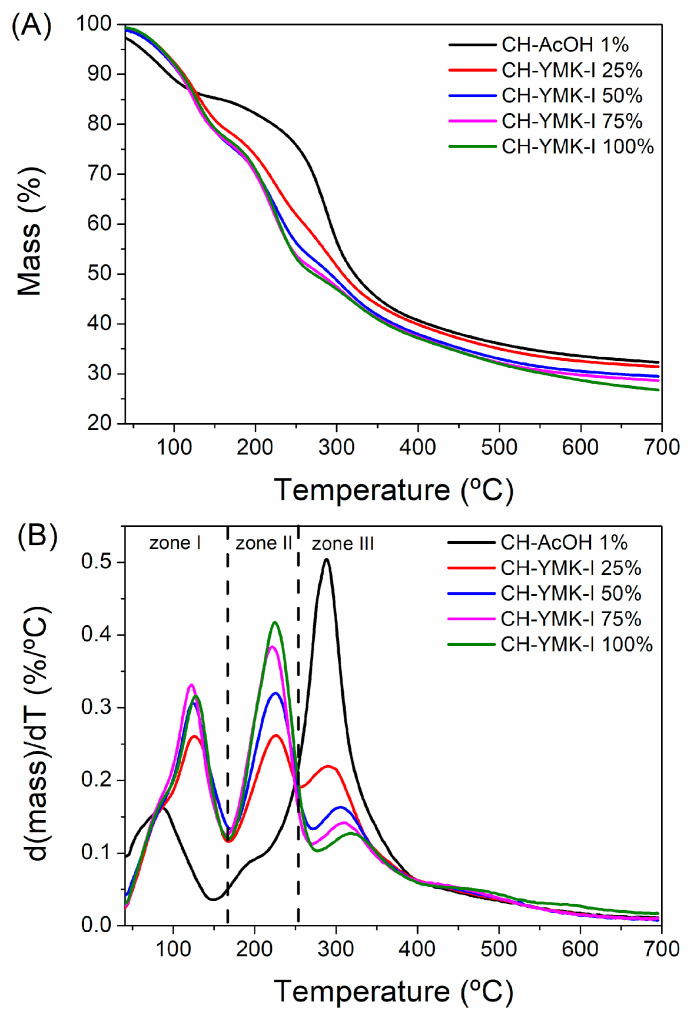
Thermogravimetric curves of chitosan and kombucha-based films: (**A**) mass loss as a function of temperature and (**B**) Derivative of the percentage mass as a function of temperature.

The results of the thermogravimetric analyses of the developed films are presented in Figure 7. The data for the initial and thermal degradation temperatures are presented in Table 4. In general, three degradation zones of mass loss were observed, depending on the composition of each sample. These zones could be observed within a temperature range from 40 to 700 °C with a residue of 30% and are associated with the decomposition of different components in the film matrix [56]. Notably, all samples showed a final residual mass of approximately 30%, suggesting the presence of thermally stable fractions, possibly due to charred biopolymeric material that remains undegraded at high temperatures [56,57,58].

The first degradation stage was observed near 100 °C across all formulations, a temperature range commonly associated with the evaporation of physically adsorbed water. This mass loss can also be partially attributed to the decomposition of low molecular weight compounds [53]. In contrast to the control films, which typically show only two major degradation zones (moisture loss and polymer backbone degradation), films incorporating the YMK-I extract exhibited a clear additional degradation stage around 225 °C. This second stage is likely due to the thermal decomposition of constituents of yerba mate infusion, since it coincides with the main peak of the infusion as showed in Figure 7B. In addition, previous studies on thermogravimetric analysis of yerba mate powder and extracts have shown similar degradation patterns in this temperature range [27]. The presence of this intermediate degradation zone in the YMK-I films confirms the successful incorporation of yerba mate-derived compounds and their impact on the thermal behavior of the film. The final degradation stage, occurring near 300 °C, is attributed to the decomposition of the primary chitosan polymer chains. This step involves the depolymerization of the biopolymer backbone, a process observed in pure chitosan and chitosan-based films in various studies [56,59].

Regarding the T_i_ values, the incorporation of YMK-I into the chitosan-based films has led to a decrease in this parameter, as the concentration of the infusion increased. This behavior suggests a reduction in the thermal stability of the films, likely due to the presence of thermolabile compounds within the infusion, such as polyphenols, organic acids or residual sugars. These components may initiate thermal decomposition at lower temperatures compared to pure chitosan. Additionally, the inclusion of the infusion may interfere with the intermolecular interactions among chitosan chains, particularly hydrogen bonding, weakening the structural integrity of the polymer matrix and leading to an earlier degradation.

#### 2.2.6. Mechanical Properties of Chitosan and Kombucha-Based Films

Mechanical properties are critical quality indicators of materials, reflecting their strength and elongation. Among these, tensile strength (TS) and elongation at break (EB) are the most used parameters to characterize chitosan-based films. These properties are closely related to the films’ homogeneity and chemical composition [60]. The mechanical performance data are presented in Table 5.

A clear trend is observed with the incorporation of YMK-I: TS values decreased significantly, while EB increased progressively and significantly with higher concentrations of the kombucha infusion. The control film (CH-AcOH 1%) showed the highest TS (41 ± 4 MPa) and the lowest EB (33 ± 4%), indicating a more rigid and less flexible structure. In contrast, films with 100% YMK-I (CH-YMK-I 100%) exhibited the lowest TS (2.0 ± 0.3 MPa) and the highest EB (274 ± 9%), suggesting a highly flexible material [61]. All variations were statistically significant (*p* < 0.05). Values of TS were similar to those described by Stefanowska et al. [23] where chitosan was dissolved in different kombucha tea and coffee infusions. However, values of EB of chitosan with YMK-I were higher than those films with tea or coffee infusion.

#### 2.2.7. Color Properties

Color is an important visual attribute for film materials, especially in applications where transparency, appearance, or color stability are critical [62]. The color parameters (L*, a*, b*) and total color difference (ΔE*) for chitosan and kombucha-based films are presented in Table 6.

The control film exhibited the highest L* value, indicating a light and almost transparent appearance, with near-neutral values in the a* and b* coordinates. As the concentration of YMK-I increased, a significant decrease in lightness (L*) was observed, with CH-YMK-I 100% reaching the lowest value, indicating darker films. The a* parameter showed a shift from slightly greenish tones (negative a* value in CH-YMK-I 25%) to reddish hues in CH-YMK-I 50–100%, with the highest a* recorded for CH-YMK-I 100%. Simultaneously, b* values increased sharply in YMK-I films, especially at 25%, reflecting a notable yellowish coloration, which remained high across all samples. The total color difference (ΔE*) values, calculated relative to the control, were substantial (ranging from 47 to 59), indicating marked visual differences. These results confirm that even small additions of YMK-I significantly alter the optical properties of the films. The color changes are likely attributed to the natural pigments and polyphenolic compounds present in the kombucha infusion, such as oxidized catechins, theaflavins and thearubigins, which may interact with the chitosan matrix [63].

#### 2.2.8. Water Vapor Permeability

In hydrophilic polymeric films characterized by continuous and homogeneous matrices, such as those studied in this work, water transport takes place via a sorption–diffusion–desorption mechanism rather than through porous pathways [64,65]. Consequently, the water vapor permeability (WVP) of these materials is governed by the hydration or solubility of water in the film, as well as the water mobility within the matrix.

Although permeability is ideally determined only by the characteristics of the polymer matrix, such as chemical structure, polarity, crystallinity, density, crosslinking degree, molecular weight, polymerization level, the presence of plasticizers and by the nature of the permeant, it is often affected by permeant concentration in real systems. In hydrophilic biopolymeric films, water molecules interact strongly with the polymer matrix, causing both water diffusivity and water solubility to vary with the partial pressure gradient across the film [64,66]. As a result, water vapor transport through hydrophilic matrices deviates markedly from ideal behavior. Moreover, the WVP of films containing hydrophilic materials such as biopolymers generally increases with film thickness due to these polymer–water interactions [64,65,67,68,69,70]. Consequently, WVP in biopolymeric films is typically reported as a single value under specific experimental conditions, which limits meaningful comparisons among films with different thicknesses.

The WVP values of the films are presented in Table 7, which also includes, as complementary information, the water flux J_w_ through the films and the corresponding film thicknesses. The control film exhibited the lowest WVP, which may be attributed to stronger intermolecular interactions between polymer chains. However, chitosan-based films contain a high number of hydroxyl and amino groups, which can readily interact with water molecules, thereby facilitating their permeation through the material [71]. The incorporation of YMK-I resulted in a progressive and statistically significant increase in WVP as the infusion concentration increased. This trend may be explained by several factors. First, the higher concentrations of YMK-I likely introduced additional hydrophilic compounds (e.g., organic acids and polyphenols) into the film matrix, which could disrupt the compact chitosan network and enhance water vapor transmission. Second, the increase in film thickness observed with higher YMK-I concentrations (from 0.038 mm in CH-AcOH 1% to 0.103 mm in CH-YMK-I 100%) may also contribute to this effect by facilitating the diffusion of water molecules. Due to the variation in film thicknesses, a direct comparison of the WVP values is not entirely appropriate, as thickness can influence the transport behavior. This behavior has been previously reported in studies on polymeric films and may be attributed to structural characteristics, such as reduced density and increased flexibility, as supported by the mechanical properties observed [64].

#### 2.2.9. Evaluation of the Antioxidant Properties by the ABST^•+^ and DPPH Assays

**Figure 8:**
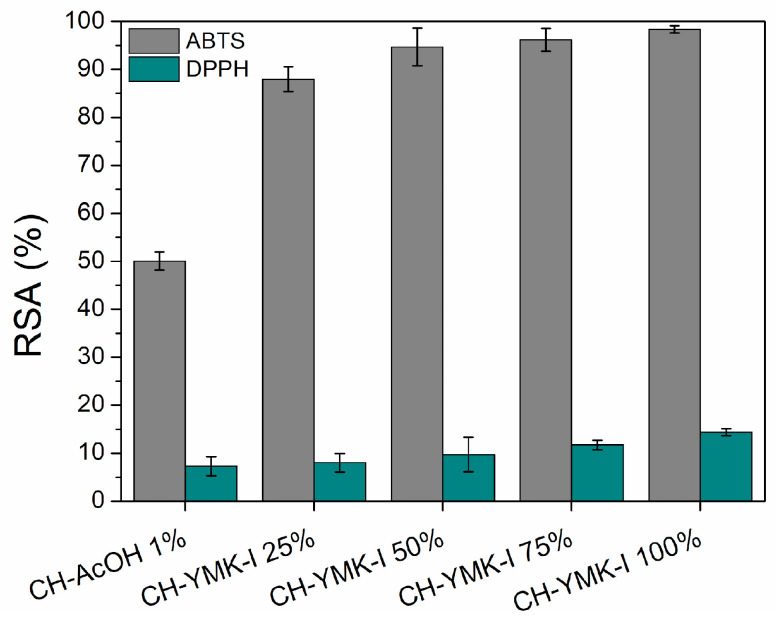
Antioxidant activity of the films by ABTS^•+^ and DPPH method.

Figure 8 shows the radical scavenging activity (%) of chitosan-based films, evaluated using the ABTS and DPPH assays. The control film exhibited low antioxidant activity, with RSA values of approximately 45% (ABTS) and 7% (DPPH), consistent with the modest intrinsic antioxidant properties of chitosan [72]. The incorporation of yerba mate kombucha infusion into the chitosan matrix led to a substantial and concentration-dependent increase in antioxidant activity, especially as measured by the ABTS assay. Films containing 25% to 100% YMK-I showed ABTS-RSA values exceeding 85%, reaching nearly 100% at the highest concentrations (75% and 100%). This enhancement can be attributed to the high content of antioxidant compounds present in YMK-I, such as polyphenols, flavonoids, and fermentation-derived metabolites, which are well-known for their radical scavenging capacity [73,74]. Same trend was observed with DPPH-RSA assay, where higher concentrations increased this value. Although the increase in RSA with higher YMK-I content is also observed in this assay, the magnitude of change is less pronounced. The lower reactivity in the DPPH assay could be due to differences in the mechanisms of the assays: ABTS is more sensitive to both hydrophilic and lipophilic antioxidants, while DPPH reacts primarily with hydrophobic molecules [75]. Given the aqueous nature of kombucha, it is plausible that most antioxidant compounds in the films are more efficiently detected by ABTS.

#### 2.2.10. Antimicrobial Activity of the Films

**Figure 9:**
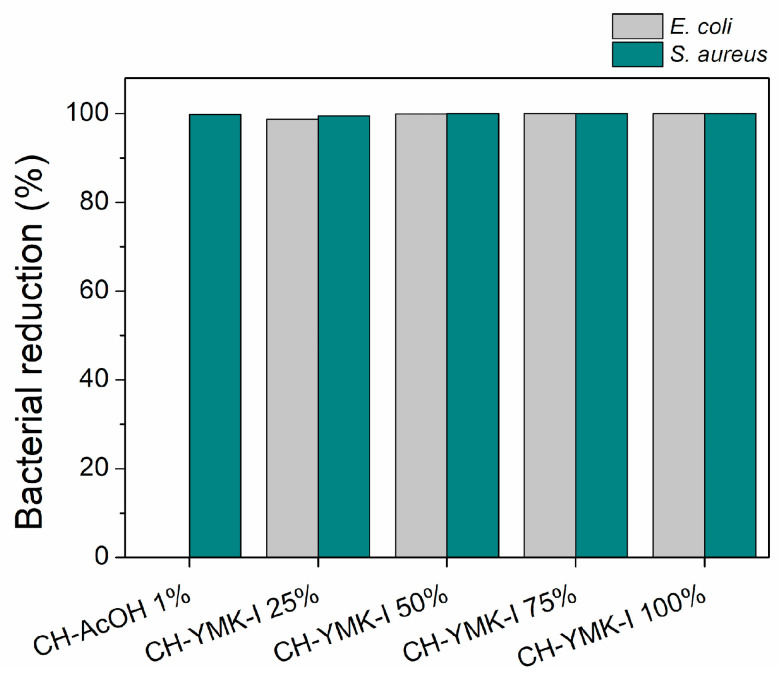
Percentage of bacterial reduction against *E. coli* and *S. aureus* of the different films.

Figure 9 illustrates the antimicrobial performance of the chitosan and kombucha yerba mate infusion films against *Escherichia coli* and *Staphylococcus aureus*. All films demonstrated high antibacterial effectiveness, with bacterial reduction values exceeding 95% for both microorganisms, regardless of YMK-I concentration.

The control film already exhibited strong antimicrobial activity against *S. aureus*, consistent with the well-documented bactericidal properties of chitosan, which acts by disrupting microbial cell membranes, chelating essential metals, and interfering with intracellular components [76]. This effect was not observed against *E. coli*, likely due to the higher resistance of Gram-negative bacteria compared to Gram-positive ones [38]. The incorporation of YMK-I at concentrations ranging from 25% to 100% further enhanced the antimicrobial activity in all cases, maintaining inhibition levels above 98%. This enhanced effect may be attributed to the acidic pH and polyphenolic compounds present in YMK-I, which could act synergistically with chitosan [77]. These components appear to be effective against both Gram-negative and Gram-positive bacteria, highlighting their potential as broad-spectrum antimicrobial agents.

## 3. Discussion

The yerba mate kombucha infusion is a strongly acidic beverage, mainly due to the presence of organic acids such as lactic and acetic acids. In addition, it contains high levels of phenolic compounds, which likely explain its antioxidant capacity and its antimicrobial activity against *S. aureus*, *P. aeruginosa*, and *E. coli*.

The use of YMK-I as a solvent for chitosan resulted in a dissolution behavior comparable to that obtained with acetic acid, as evidenced by rheological analysis. Once the films were formed, FTIR-ATR analysis revealed interactions between the compounds present in the infusion and the functional groups of chitosan, indicating good compatibility between components and a homogeneous system.

The incorporation of YMK-I significantly influenced the thermal behavior of the films. On one hand, it acted as a plasticizer, increasing chain mobility and flexibility. On the other hand, it modified the thermal degradation profile by introducing an additional degradation event associated with the thermolabile compounds present in the infusion. Consequently, a decrease in thermal stability was observed, which can be attributed both to the presence of these compounds and to the partial disruption of intermolecular interactions within the polymer matrix. However, this reduction does not compromise the practical applicability of the materials, as they retain sufficient thermal stability within typical processing and usage conditions, such as those required in food packaging, biomedical, or cosmetic applications.

Mechanical properties further supported the plasticizing effect of YMK-I, as its incorporation reduced intermolecular interactions and increased the mobility of chitosan chains. This behavior is consistent with that of conventional plasticizers, which are small molecules that reduce brittleness and improve flexibility and processability. In this context, the components of the kombucha infusion likely interact with the polymer network, facilitating chain movement and softening the structure. These findings highlight the dual role of YMK-I as both a functional additive and a natural plasticizer in the development of flexible chitosan-based films.

In terms of optical properties, the incorporation of the infusion led to noticeable color changes in the films. These modifications may be advantageous or limiting depending on the intended application. For example, darker films could be beneficial for packaging light-sensitive products, whereas more transparent films may be preferred when product visibility is required.

Regarding barrier properties, the addition of YMK-I increased water vapor permeability (WVP), which is consistent with previous studies reporting that plant-based extracts can alter barrier performance by partially disrupting polymer–polymer interactions and introducing hydrophilic groups into the matrix.

Finally, the antioxidant capacity of the films was significantly enhanced by the incorporation of YMK-I, reinforcing their potential use in active food packaging applications aimed at delaying oxidative processes. Overall, these results confirm that chitosan-based films incorporating fermented beverages such as YMK-I can combine antimicrobial and antioxidant functionalities, making them promising materials for advanced packaging systems.

## 4. Materials and Methods

### 4.1. Materials

Chitosan (Biorigin^®^, São Paulo, Brazil), sodium hydroxide (NaOH, ≥98%, Sigma-Aldrich^®^, Darmstadt, Germany), potassium carbonate (K_2_CO_3_, Sigma-Aldrich^®^), magnesium nitrate (Mg(NO_3_)_2_, 98%, Sigma-Aldrich^®^), barium chloride (BaCl_2_, ≥98%, Sigma-Aldrich^®^), phosphate-buffered saline (PBS, pH 7.4, Sigma-Aldrich^®^), 2,2′-azino-bis(3-ethylbenzothiazoline-6-sulfonic acid) diammonium salt (ABTS, Sigma^®^, Tokyo, Japan), potassium persulfate (K_2_S_2_O_8_, Sigma^®^), 2,2-diphenyl-1-picrylhydrazyl (DPPH, Sigma^®^), peptone (Britania^®^, Kolkata, India), bacteriological agar (Britania^®^), nutrient broth (Britania^®^). Analytical-grade organic acid standards (lactic and acetic acids) were purchased from (Sigma-Aldrich^®^). Ultrapure water (Milli-Q, Millipore, Burlington, MA, USA) was used throughout, and samples were filtered through 0.45 µm nylon syringe filters prior to HPLC analysis. Yerba mate used in this work, was a commercial yerba mate “La Tranquera” (J. Llorente and Cía S.A., Oberá, Misiones, Argentina).

### 4.2. Preparation of Yerba Mate Kombucha Infusion (YMK-I)

The infusion was prepared by steeping 6 g/L of yerba mate in water at 80 °C for 10 min. Following extraction, the plant material was removed and sucrose was added at a concentration of 100 g/L. Fermentation was initiated by inoculating the infusion with a starter culture obtained from the LOMCEM laboratory at an inoculum-to-infusion ratio of 1:5, reaching a final volume of 1.5 L. The culture was incubated under static conditions at 22 °C in a 3 L glass vessel covered with cellulose cloth for a period of 21 days. Upon completion of the fermentation process, the obtained fermented infusion (YMK-I) was used for the preparation of active chitosan films. Prior to use, YMK-I was filtered and sterilized in an autoclave at 121 °C for 15 min under a pressure of 1 atm.

### 4.3. Characterization of YMK-I

#### 4.3.1. Determination of pH, Titratable Acidity and Total Solids

The pH was measured using a pH meter (Altronix TPA-V, SAEN SRL, Buenos Aires, Argentina). Titratable acidity (TA) was determined by acid–base titration, in which the kombucha infusion was titrated with standardized 0.1 M sodium hydroxide to pH 8.2, using phenolphthalein as the indicator.

The calculated TA was expressed as a percentage (%), grams of acetic acid per 100 mL of sample, as it is the major organic acid in kombucha (Equation (1)).


(1) $$\%\;Titratable\;acidity\;of\;acetic\;acid=\frac{V_{NaOH}\times M_{NaOH}\times 60.053\times 0.001}{V_{sample}}\times 100$$

V_NaOH_ = volume of NaOH (mL)

M_NaOH_ = molarity of NaOH (M)

V_sample_ = volume of sample (mL)

The total solids content was determined gravimetrically. Kombucha infusion samples collected after fermentation were filtered through Whatman No. 4 filter paper. The filtrate was dried in an oven at 105 °C to constant weight, and results were expressed as grams of solids per 100 mL of infusion (%).

#### 4.3.2. Determination of Total Phenolic Content

Total phenolic content was determined using an adapted Folin–Ciocalteu colorimetric assay described by Rezzani et al. (2025) [78]. Briefly, 250 µL of YMK-I were combined with 1250 µL of Folin–Ciocalteu reagent (previously diluted 1:10) and 1000 µL of sodium carbonate solution (7.5% *w*/*v*). The reaction mixture was incubated in the absence of light for 2 h. Absorbance readings were recorded at 765 nm using a UV–visible spectrophotometer (UV-9000S, Shanghai, China). All measurements were performed in triplicate, and results were reported as milligrams of gallic acid equivalents (GAE) per liter of YMK-I.

#### 4.3.3. Determination of Reducing Sugars

The determination of reducing sugars was carried out using the 3,5-dinitrosalicylic acid (DNS) method, a commonly applied colorimetric technique [36]. This assay is based on the oxidation of the aldehyde group of reducing sugars to their corresponding acids, while DNS is reduced to 3-amino-5-nitrosalicylic acid in an alkaline medium. Upon completion of the reaction, a characteristic red-brown coloration develops, which is measured spectrophotometrically at 540 nm. The concentration of total reducing sugars was calculated using a calibration curve and expressed as milligrams of glucose per liter of YMK-I.

#### 4.3.4. High-Performance Liquid Chromatography (HPLC)

Determination of short-chain organic acids was performed by high-performance liquid chromatography using a Beckman System Gold chromatographic system (Beckman Coulter, Brea, CA, USA), equipped with a 125 solvent delivery module and a 166 UV–Vis detector. The system was operated and controlled by Karat 32 chromatography software 8.0 (Beckman Coulter), which was used for instrument control, data acquisition, and chromatographic data processing.

For this purpose, an aliquot of 20 µL of diluted and filtered sample was injected into the system. Chromatographic separation was achieved using an ion-exclusion column (Hamilton PRP-X300, 7 µm; 250 × 4.1 mm; Hamilton, Reno, NV, USA), employing 1 mN sulfuric acid in HPLC grade water as the mobile phase at a flow rate of 2.0 mL min^−1^ and ambient temperature. Detection was carried out at 210 nm. Quantification was performed by external calibration using standard solutions of the corresponding organic acids at a concentration of 0.2 mg mL^−1^. All determinations were performed in triplicate.

#### 4.3.5. Minimum Inhibitory Concentration (MIC)

Minimum inhibitory concentrations (MICs) were determined using the broth microdilution method against the Gram-positive bacterium *Staphylococcus aureus* (ATCC 25923) and the Gram-negative bacteria *Escherichia coli* (ATCC 25922) and *Pseudomonas aeruginosa* (ATCC 9027), obtained from Thermo Scientific™ (Waltham, MA, USA), following the guidelines established by the Clinical and Laboratory Standards Institute (CLSI) [79]. Initially, bacterial suspensions were prepared in sterile 0.1% peptone water at a concentration of approximately 10^8^ colony-forming units (CFU)/mL, adjusted to a turbidity equivalent to a 0.5–1.0 McFarland standard. Prior to this, bacterial isolates were cultured on nutrient agar and incubated at 37 °C for 24 h. The suspensions were subsequently diluted 1:100 in Mueller–Hinton broth to obtain a final inoculum concentration of approximately 10^6^ CFU/mL. For the assay, 200 μL of YMK-I were added to the first column of a sterile 96-well microplate, while 100 μL of Mueller–Hinton broth were dispensed into the remaining wells, excluding the first column. Serial twofold dilutions of YMK-I were then prepared across the plate, except for the final column. Subsequently, 100 μL of each bacterial suspension were added to all wells, resulting in a final volume of 200 μL per well. The last column, containing no YMK-I, served as the positive growth control. The microplates were incubated at 37 °C for 24 h, after which the MIC value was visually determined as the lowest YMK-I concentration capable of inhibiting visible microbial growth.

### 4.4. Preparation of Chitosan and Kombucha-Based Films

For film preparation, chitosan solutions at 1.5% *w*/*w* were prepared using YMK-I as a solvent. These solutions were formulated at 25%, 50%, 75%, and 100% *v*/*v* YMK-I in distilled water. Additionally, a control sample was prepared using 1% *v*/*v* acetic acid in distilled water. The final solutions were mixed using a mechanical stirrer for 24 h at room temperature. For the preparation of chitosan–kombucha films, 15 g of each film-forming solution were cast into Petri dishes with a diameter of 90 mm and dried at 40 °C for 16 h. After drying, the films were carefully removed from the dishes and conditioned for one week at 25 °C under 43% relative humidity, provided by a saturated K_2_CO_3_ solution, before further characterization. The nomenclature and corresponding descriptions of the prepared films are summarized in Table 8. As previously indicated, films prepared without YMK-I (CH–AcOH 1%) were used as control samples.

#### Rheological Evaluation of Film-Forming Dispersions

The rheological behaviour of chitosan dissolved in acetic acid dilutions and in various dilutions of yerba mate kombucha tea was studied using rotational rheometry. The acetic acid and yerba mate kombucha tea dilutions used to dissolve chitosan were also analysed as controls. Rheological behavior was characterized by obtaining flow curves using an AR-G2 rheometer (TA Instruments, New Castle, DE, USA) equipped with a 40 mm diameter steel cone-plate geometry (2° angle) and a truncation gap of 55 μm. Measurements were carried out in triplicate at 22 °C using 0.7 mL of sample. Shear rates were progressively increased within a range of 0.005–1000 s^−1^. The relationship between shear stress (τ, Pa) and shear rate (γ, s^−1^) was determined, and the resulting flow curves were fitted using the Herschel–Bulkley model according to Equation (2). In this model, τ_0_ (Pa) corresponds to the yield stress, defined as the stress required to initiate flow at zero shear rate; K represents the consistency index, which is related to the apparent viscosity of the dispersion [80,81]; and n is the flow behavior index, describing deviations from Newtonian behavior (n > 1 for dilatant fluids and n < 1 for pseudoplastic fluids). Model fitting of the experimental data was performed using OriginPro 8 software (OriginLab Corporation, Northampton, MA, USA).


(2) $$\tau =\tau_{0}+K\;\gamma^{n}$$

### 4.5. Characterization of Active Films

#### 4.5.1. Attenuated Total Reflectance Fourier Transform Infrared Spectroscopy (ATR-FTIR)

FTIR analysis was carried out for all film samples using an IR Affinity-1 Shimadzu spectrophotometer (Shimadzu Co., Kyoto, Japan) equipped with an attenuated total reflectance (ATR) accessory featuring a diamond crystal (GladiATR, Pike Technologies, Madison, WI, USA). Spectra were recorded over a wavenumber range of 400–4000 cm^−1^ using 48 scans at a resolution of 4 cm^−1^ and applying Happ-Genzel apodization. All measurements were performed in duplicate.

#### 4.5.2. Scanning Electron Microscopy (SEM)

The microstructural characteristics of the films were examined through observation of both surface and cross-sectional morphologies using a FEI-Quanta 200 scanning electron microscope (FEI Co., Hillsboro, OR, USA) operating at 5 kV. Cross-sectional samples were prepared by cutting the films with a sterile surgical blade at room temperature. Prior to analysis, specimens were mounted on sample holders and maintained at 22 °C and 43% relative humidity. All samples were sputter-coated with a thin gold layer before imaging. Surface and cross-sectional micrographs were collected at a magnification of 1500× under high-vacuum conditions.

#### 4.5.3. Thermal Properties

The determination of the thermal properties of the infusion and the films, were accomplished by differential scanning calorimetry (DSC), using a calorimeter (TA Instruments Q200, New Castle, DE, USA) and Thermogravimetry analysis (TGA), using a thermobalance (Q-500 thermogravimetric analyzer, TA Instruments, New Castle, DE, USA). DSC analyses of the infusion, a volume of 30 µL of YMK-I (approximately 2 mg) was placed into aluminium pans, which were then introduced into a desiccator to allow water evaporation. After drying, the samples were hermetically sealed and heated from −50 °C to 160 °C at a rate of 10 °C min^−1^. While for the film samples, each dried film was sealed into aluminium pans with hermetic lids and heated from −80 °C to 180 °C at a rate of 10 °C min^−1^. All samples were analysed in duplicates and thermal transitions were determined using TA Universal Analysis software (v4.5, TA Instruments, New Castle, DE, USA). In addition, the residual solids present in the infusion were characterized by thermogravimetric analysis (TGA) using a Q-500 thermogravimetric analyzer (TA Instruments, New Castle, DE, USA). Approximately 15 mg of the infusion were weighed and heated from 40 °C to 700 °C at a rate of 10 °C min^−1^ under a nitrogen atmosphere, with flow rates of 40 mL min^−1^ for the balance and 60 mL min^−1^ for the sample chamber. For film characterization, approximately 7 mg of each sample were analyzed under the same experimental conditions used for the infusion samples. From the TGA curves, the initial degradation temperature (Ti) was determined at 10% mass loss, while the maximum degradation temperature (Tmax) for each degradation stage was obtained from the derivative thermogravimetric (DTG) curves corresponding to mass loss as a function of temperature. All measurements were performed in duplicate.

#### 4.5.4. Mechanical Properties

Mechanical properties were evaluated at room temperature using a Universal Testing Instrument (Megatest TC-500 Series II, Megatest, Buenos Aires, Argentina) equipped with a 30 kgf load cell. Measurements were carried out at a crosshead speed of 15 mm min^−1^ with an initial gauge separation of 25 mm. Prior to analysis, rectangular specimens (50 mm × 10 mm) from each formulation were prepared and conditioned at 53% relative humidity using a saturated Mg(NO_3_)_2_ solution. Film thickness was measured at five different locations on each specimen using a digital micrometer (INSIZE Co., Ltd, Suzhou New District, Suzhou, China; ±0.001 mm accuracy). Mechanical parameters, including Young’s modulus (YM, MPa), tensile strength (TS, MPa), and elongation at break (EB, %), were obtained from the corresponding stress–strain curves.

#### 4.5.5. Color Properties

The visual characteristics of the films were evaluated by measuring CIELab color parameters using a Konica Minolta CR400 spectrophotometer (Tuscaloosa, NJ, USA). The parameter L represents lightness or darkness, a indicates the color range from green to red (−a* corresponding to greener tones and +a* to redder tones), and b* describes the variation from blue to yellow (−b* indicating bluer shades and +b* yellower shades). In addition, the total color difference (ΔE) was determined according to Equation (3).

(3) $$∆E=\sqrt{{\left(a^{*}-a_{0}^{*}\right)}^{2}+{\left(b^{*}-b_{0}^{*}\right)}^{2}+{\left(L^{*}-L_{0}^{*}\right)}^{2}}$$

where a_0_*, b_0_*, and L_0_* are the coordinates corresponding to the control film, against which the rest of the films were compared in order to determine the color change owing to the incorporation of YMK-I.

#### 4.5.6. Water Vapor Permeability (WVP)

Water vapor permeability of the films was determined by quantifying the water vapor flow through the films gravimetrically, following the ASTM E-E96 [82] standard with some modifications [65]. Firstly, the films were placed in acrylic cells containing a saturated solution of BaCl_2_, which provides inside the cell a r.h. of 90%. The cells were placed inside a desiccator containing a saturated solution of NaOH to provide a r.h. of 10%, at a constant temperature of 22 °C. A fan was placed over the films to maintain uniform air conditions inside the desiccator, following previous recommendations from previous authors [65]. The weights loss of the cells (m_H2O_), indicating the amount of water transported through the films, were recorded at specified time intervals using a precision analytical balance (Precisa 125 A SCS, 10^−3^ g). m_H2O_ was plotted as a function of time (t), and after reaching steady-state conditions, as indicated by a linear relationship, measurements were continued for an additional 30 h. Water vapor flux (J_w_) was calculated from the slope (Δm_H2O_/Δt) obtained by linear regression of sample weight loss versus time, according to Equation (4).

(4) $$J_{w}=\frac{1}{A}\left(\frac{{∆m}_{H2O}}{∆t}\right)$$

where A is the effective area of the exposed film. WVP was calculated by Equation (5)

(5) $$WVP=\frac{J_{w}L}{{∆p}_{w}}$$

where L is the film thickness and Δp_w_ = (p_w2_ − p_w1_) (in Pa units) is the differential water vapor partial pressure across the film; p_w1_ is the partial pressure of water vapor at the film surface outside the cup (263.9 Pa); and p_w2_ is the partial pressure of water vapor at the film surface inside the cup (2375.4 Pa). Experiments were performed in duplicate.

#### 4.5.7. Antioxidant Properties

The antioxidant activity of the films was assessed using both ABTS and DPPH assays. For the ABTS analysis, the ABTS^•+^ radical cation was generated in an aqueous medium by combining 7 mM ABTS with 2.45 mM K_2_S_2_O_8_ (solution A). The mixture was kept in the dark for 16 h to allow radical formation. Subsequently, solution A was diluted with Milli-Q water (solution B) until an absorbance value of 0.70 ± 0.02 at 734 nm was obtained. Film samples (approximately 8 mg) were cut into discs and placed in Eppendorf tubes containing 1 mL of solution B. After incubation for 5 min in the dark under gentle agitation, the absorbance was measured at 734 nm. All experiments were conducted at least in duplicate.

Furthermore, antioxidant activity was also determined using the DPPH radical assay, since different antioxidant tests may identify distinct mechanisms of action. Therefore, applying both methodologies provided a broader characterization of the antioxidant properties of the films. The DPPH method is based on the reduction in the stable DPPH radical through hydrogen donation from antioxidant compounds, leading to a decrease in color intensity. A 1 mM DPPH solution was prepared in absolute ethanol and allowed to react with the samples for 1 h in darkness under gentle agitation. The absorbance was then measured at 517 nm, and all analyses were carried out at least in duplicate.

The antioxidant activity of the different films, by using both methods, was calculated by the radical scavenging activity percentage (RSA(%)), as described in Equation (6).

(6) $$RSA(\%)=\frac{blank\;absorbance-sample\;absorbance}{blank\;absorbance}\times 100$$

where blank corresponds to ABTS solution B absorbance without film.

#### 4.5.8. Antimicrobial Activity

The antimicrobial properties of the films were assessed following a procedure adapted from ASTM E2149-01 [83]. The evaluation was performed against both Gram-negative and Gram-positive bacterial strains, namely *Escherichia coli* (ATCC 25922) and *Staphylococcus aureus* (ATCC 25923), respectively. The bacterial strains were first cultured on sterile nutrient agar plates and incubated at 37 °C for 24 h. Subsequently, bacterial suspensions were prepared in sterile PBS (pH 7.4) to achieve a final concentration of 10^5^ CFU/mL.

Films were then cut into 1 cm^2^ pieces and placed into Eppendorf tubes containing 1 mL of bacterial suspension. Samples were incubated at 37 °C under continuous shaking at 100 rpm. Aliquots were collected at the beginning of the experiment (0 h) and after 24 h of contact, followed by total viable cell determination. A bacterial suspension without film was used as a control, and films lacking YMK-I were also included as control samples. The percentage of bacterial reduction was calculated according to Equation (7).

(7) $$Bacterial\;reduction\;\left(\%\right)=\frac{B-A}{B}\times 100$$

where A is CFU/mL for the film after 24 h contact time, and B is CFU/mL at the initial time.

### 4.6. Statistical Analysis

The results are presented as mean values ± standard deviation (S.D.). Statistical analysis was performed using analysis of variance (ANOVA) with PSPP software 2.1.1. Differences among mean values were evaluated using Tukey’s post hoc test, considering statistical significance at *p* < 0.05.

## 5. Conclusions

In conclusion, results obtained in this work support the use of YMK-I not only as an effective acidic medium for dissolving chitosan, but also as natural plasticizer and functional component with potential antioxidant and antimicrobial properties for the development of bioactive materials. Its dual role as both a solvent and an active ingredient highlights its added value in material design, particularly in applications where antimicrobial and antioxidant performance is desirable, such as food packaging, biomedical coatings, or controlled-release systems. Therefore, YMK-I represents a promising alternative for the formulation of multifunctional chitosan-based materials with enhanced technological and biological performance.

## Figures and Tables

**Table 1:** Organic acids detected in the kombucha infusion and their concentrations determined by HPLC.

Organic Acid	Concentration (mg/L)	Retention Time
Lactic	194	3.275
Acetic	909	4.188

**Table 2:** Values of the parameters fitted for flow curves of Figure 2 by using the Herschel–Bulkley model. Errors of parameters were estimated from the fit analysis.

	Samples	Herschel–Bulkley Model			
		*τ*_0_ (10^−2^ Pa)	*K* (10^−4^ Pa.s)	*n*	*R* ^2^
Dilutions	AcOH 1%	13 ± 7	9.0 ± 0.6	1.02 ± 0.01	0.999
	YMK-I 25%	5 ± 3	10.0 ± 0.2	1.00 ± 0.01	0.999
	YMK-I 50%	16 ± 9	9.8 ± 0.8	1.02 ± 0.01	0.999
	YMK-I 75%	12 ± 8	11.0 ± 0.7	1.01 ± 0.01	0.999
	YMK-I 100%	12 ± 9	11.8 ± 0.8	1.01 ± 0.01	0.999
Chitosan dissolutions	CH-AcOH 1%	0.0 ± 0.2	889 ± 22	0.91 ± 0.01	0.999
	CH-YMK-I 25%	0.0 ± 0.2	786 ± 17	0.92 ± 0.01	0.999
	CH-YMK-I 50%	0.0 ± 0.2	805 ± 18	0.92 ± 0.01	0.999
	CH-YMK-I 75%	0.0 ± 0.2	802 ± 17	0.92 ± 0.01	0.999
	CH-YMK-I 100%	0.0 ± 0.2	859 ± 19	0.92 ± 0.01	0.999

**Table 4:** Temperatures correspond to the initial and maximum degradation temperatures of chitosan and kombucha-based films. Temperature error: ±1 °C.

Sample	T_i_ (°C)	T_max1_ (°C)	T_max2_ (°C)	T_max3_ (°C)
CH-AcOH 1%	158	87	291	--
CH-YMK-I 25%	132	126	227	289
CH-YMK-I 50%	125	124	226	306
CH-YMK-I 75%	124	123	221	310
CH-YMK-I 100%	128	128	225	318

**Table 5:** Mechanical parameters of chitosan and kombucha-based films.

Sample	TS (MPa)	EB (%)
CH-AcOH 1%	41 ± 4 ^a^	33 ± 4 ^a^
CH-YMK-I 25%	26 ± 2 ^b^	51 ± 4 ^b^
CH-YMK-I 50%	3.5 ± 0.4 ^c^	95 ± 3 ^c^
CH-YMK-I 75%	3.2 ± 0.3 ^c^	178 ± 5 ^d^
CH-YMK-I 100%	2.0 ± 0.3 ^d^	274 ± 9 ^e^

The same letters in the data reported in a column mean non-significant differences (*p* ≥ 0.05).

**Table 6:** Color properties of chitosan and kombucha-based films.

Sample	L*	a*	b*	ΔE*
CH-AcOH 1%	90 ± 1 ^a^	0.97 ± 0.06 ^a^	0.63 ± 0.32 ^a^	-
CH-YMK-I 25%	81 ± 2 ^b^	−4.9 ± 1.7 ^b^	59 ± 5 ^b^	59
CH-YMK-I 50%	76 ± 1 ^c^	2.2 ± 0.3 ^c^	45 ± 1 ^c^	47
CH-YMK-I 75%	71 ± 3 ^cd^	6.3 ± 2.3 ^d^	52 ± 3 ^b^	56
CH-YMK-I 100%	69 ± 2 ^d^	8.3 ± 1.4 ^d^	55 ± 2 ^b^	59

The same letters in the data reported in a column mean non-significant differences (*p* ≥ 0.05).

**Table 7:** Water flux J_w_, water vapor permeability values of chitosan and kombucha-based films.

Sample	J_w_ (10^−5^ g s^−1^ m^−2^)	Permeability (10^−10^ g s^−1^ m^−1^ Pa^−1^)	Thickness (mm)
CH-AcOH 1%	737 ± 3	1.31 ± 0.003	0.038 ± 0.008
CH-YMK-I 25%	455 ± 3	1.40 ± 0.005	0.059 ± 0.011
CH-YMK-I 50%	480 ± 38	2.48 ± 0.098	0.084 ± 0.003
CH-YMK-I 75%	538 ± 1	3.23 ± 0.016	0.105 ± 0.003
CH-YMK-I 100%	584 ± 2	3.60 ± 0.002	0.103 ± 0.001

**Table 8:** Description of chitosan and kombucha-based films.

Sample	Description
CH-AcOH 1%	Films based on chitosan and 1% *v*/*v* of acetic acid in distilled water
CH-YMK-I 25%	Films based on chitosan and 25% *v*/*v* of YMK-I in distilled water
CH-YMK-I 50%	Films based on chitosan and 50% *v*/*v* of YMK-I in distilled water
CH-YMK-I 75%	Films based on chitosan and 75% *v*/*v* of YMK-I in distilled water
CH-YMK-I 100%	Films based on chitosan and 100% *v*/*v* of YMK-I in distilled water

## Data Availability

The original contributions presented in this study are included in the article. Further inquiries can be directed to the corresponding authors.

## Abbreviations

The following abbreviations are used in this manuscript:

YMK-I	Yerba Mate Kombucha Infusion
HPLC	High Performance Liquid Chromatography
MIC	Minimum Inhibition Concentration
RSA	Radical Scavenging Activity
FTIR-ATR	Fourier Transformed Infrared with Attenuated Total Reflectance
DSC	Differential Scanning Calorimetry
TGA	Thermogravimetric analysis
CH	Chitosan
